# Bi-columnar locking plate fixation through a combined medial and lateral approach for the treatment of low transcondylar fractures of the distal humerus in the elderly

**DOI:** 10.1186/s12891-022-05594-1

**Published:** 2022-08-11

**Authors:** Sam-Guk Park, Hyun-Gyu Seok

**Affiliations:** Department of Orthopedic Surgery, Yeungnam University Medical Center, Yeungnam University Hospital, Yeungnam University College of Medicine, 170 Hyeonchung-ro, Nam-gu, Daegu, 42415 Korea

**Keywords:** Humerus, Distal, Low transcondylar, Fracture, Medial and lateral approach

## Abstract

**Background:**

Low transcondylar fractures (LTFs) of the distal humerus are relatively uncommon elbow injuries in elderly patients after low-energy injuries. Although there is still debate regarding the method of fixation, several surgeons prefer bi-columnar fixation using pre-contoured locking plates. However, posterior approaches, which are usually used to perform the above procedure, have disadvantages, such as ulnar nerve neuropathy, damage to the extensor mechanism, and the need for general anesthesia. To solve these problems, the authors designed a combined medial and lateral approach. The purpose of this study was to present the outcomes of bi-columnar internal fixation through a combined medial and lateral approach for the treatment of LTFs of the distal humerus in the elderly.

**Methods:**

A total of 46 patients diagnosed with distal humeral fractures between May 2017 and April 2020 were included. Thirty patients were excluded, and 16 patients who underwent open reduction and internal fixation by the medial and lateral approach were selected. We carried out all the surgeries under brachial plexus anesthesia. The clinical outcomes were assessed based on the visual analog scale (VAS) score, Mayo elbow performance score (MEPS), disabilities of the arm, shoulder, and hand (DASH) score, and range of motion (ROM) of the elbow joint. Standardized radiographs were obtained at 3, 6, and 12 months after surgery and at the last follow-up visit to evaluate for bony union and to check for complications, such as ulnar nerve neuropathy and heterotopic ossification.

**Results:**

The mean age was 81 years (range, 65–91 years). Bony union was achieved in 15 out of 16 patients. The mean VAS score was 2.1 (range, 0–6), the mean MEPS was 84.4 (range, 70–100), and the mean DASH score was 20.6 (range, 9.5–33.6). There were three complications including reduction loss, skin necrosis, and stiffness of the elbow. There was no ulnar nerve neuropathy. The post-operative ROM was 100 degrees or higher in all cases, which did not cause any impairment in daily life.

**Conclusion:**

LTFs of the distal humerus in the elderly can yield satisfactory results with bi-columnar internal fixation through a combined medial and lateral approach.

## Background

Low transcondylar fractures (LTFs) of the distal humerus are relatively uncommon elbow injuries. The majority of surgeons believe that operative treatment of LTFs in the elderly is particularly challenging. Low transverse, intra-capsular, and extra-articular fracture lines running across the olecranon and coronoid fossae characterize these fractures. Such fractures tend to occur in elderly patients after low-energy injuries and are uncommon after acute trauma in young patients [1–3]. Due to the complexity of distal humeral geometry and presence of small and osteoporotic distal fragments, stable internal fixation may be extremely difficult to achieve [4].

The principle of non-surgical treatment is limited to patients who cannot tolerate anesthesia. With the emergence and rapid development of the semi-constrained elbow prosthesis, total elbow joint replacement has gradually become an important treatment option, particularly in individuals engaged in low physical demand activities and with fractures not amenable to plate fixation [5]. However, total elbow arthroplasty has several limitations. Notably, patients who undergo total elbow arthroplasty must follow lifelong stringent weight restrictions. Additionally, relatively critical complications, such as aseptic loosening and periprosthetic fractures may occur.

Currently, bi-columnar anatomic locking plate fixation is the standard of care for most individuals with LTF and has been shown to provide more predictable outcomes and earlier joint mobilization [6–8].

Despite these advances in treatment, open reduction and internal fixation (ORIF) yields a fair number of complications. In particular, postoperative ulnar nerve changes, nonunion of the olecranon, removal of the olecranon hardware, and breakdown in the thin posterior elbow skin are inherent complications with the posterior approach. A recent systematic review has shown that ORIF for distal humerus fractures in the elderly population has an overall complication rate of 30% due to complications such as operative ulnar nerve change (7.2%), olecranon osteotomy nonunion (2.7%), and superficial wound problems (6.9%) [9]. Pre-existing chronic diseases in elderly patients can further complicate general anesthesia for the conventional elbow posterior approach in the prone or lateral positions.

Xie et al. [10] presented a combined medial and lateral approach to treat intra-articular distal humerus fractures. We have treated patients with LTF with bi-columnar locking plate fixation through this approach. We were able to carry out all the surgeries under brachial plexus anesthesia without violating the elbow extension mechanism and without ulnar nerve dissection. In the present study, we report the results of 16 elderly patients with LTF, who underwent open reduction and bi-columnar anatomic locking compression plate (LCP) fixation through a combined medial and lateral approach.

## Methods

After approval of the study protocol by the institutional review board, patients diagnosed with distal humeral fractures between May 2017 and April 2020 were retrospectively included in this study. The patient records and serial radiographs were assessed. The medical charts of all patients were reviewed; subsequently, data regarding the following were retrieved: age, sex, trauma mechanism, operation time, blood loss, other injuries besides the humeral shaft fracture, type of fracture, and union time.

The following inclusion criteria were applied: (1) low transverse type of distal humeral fractures (type A2.3 and type A3 according to the Arbeitsgemeinshaft Osteosynthesfragen/Orthopaedic Trauma Association (AO/OTA) classification); (2) patients of age 65 years or older; (3) follow-up for at least 12 months after surgery. The exclusion criteria were as follows: (1) polytrauma; (2) pathologic fractures; (3) periprosthetic fractures; (4) severe arthritis or inflammatory arthropathy of the elbow joint; (5) fractures involving the articular surface of the distal humerus; and (6) incomplete follow-up data.

**Fig. 1:**
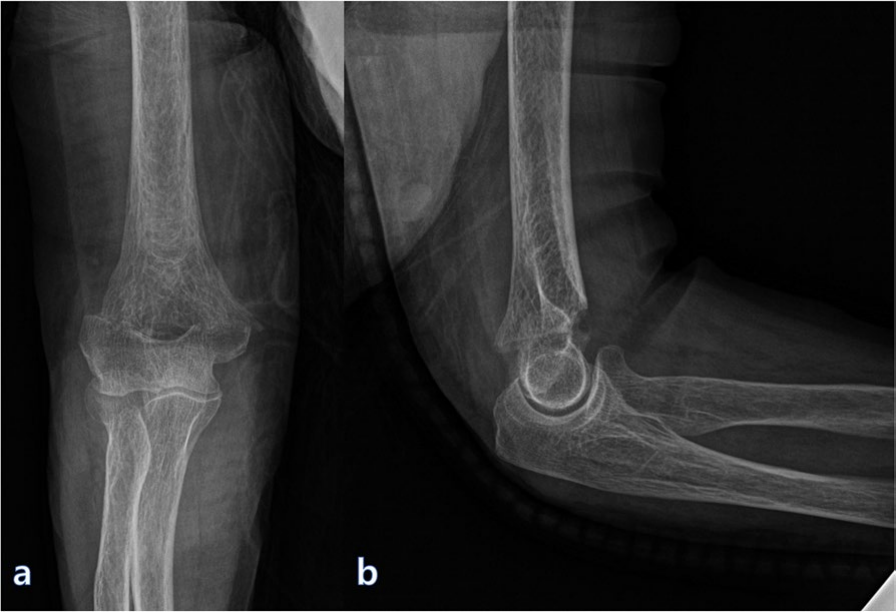
**a** Anterior–posterior and **b** lateral radiographs of a 75-year-old female patient taken before surgery. The fracture was defined as an extra-articular fracture with a single transverse fracture line that consistently exited at the level of or distal to the lateral epicondyle laterally and at the level of or just proximal to the medial epicondyle

LTF was defined as an extra-articular fracture with a single transverse fracture line that consistently exited at the level of or distal to the lateral epicondyle laterally and at the level of or just proximal to the medial epicondyle (Fig. 1). None of the fractures included in the study extended proximal to the roof of the olecranon fossa affecting the columns. All the patients underwent a computed tomography (CT) scan prior to surgery. Radiologic classification was carried out using the AO system, which is identical to the OTA classification for distal humeral fractures. We selected only patients with type A2.3 and type A3 LTFs according to the AO/OTA classification. Radiological consolidation was defined as cortical bridging of at least three out of four cortices and was expressed in weeks from the day of the fracture. Delayed union was defined as a failure to heal at 24 weeks post-fracture, with no progress toward healing as seen on the most recent radiographs. Nonunion was defined as no evidence of bone union at 1 year after injury. Information regarding the affected side, consolidation period, and presence of delayed union were collected from the radiographs and hospital records of the patient.

We identified 46 consecutive distal humeral fractures through our registry. Two individuals reviewed all the radiographs to determine the number of fractures that fulfilled the previously mentioned criteria and excluded 30 patients from the study. Of the 30 patients, 13 patients had polytrauma and 5 had fractures involving intra-articular surfaces. Twelve patients were excluded due to incomplete follow-up data. Ultimately, 16 patients were included in this study.

## Surgical technique

### Patient positioning and anesthesia

All operations were performed by a single surgeon, with patients under brachial plexus anesthesia, occasionally accompanied by moderate sedation. The patient was placed in the supine position, and the arm to be operated on was positioned at 90° of abduction on a radiolucent operating table. A sterile pneumatic tourniquet was applied as proximally as possible on the arm. A small, rolled towel was placed under the ipsilateral wrist to keep the elbow flexed at approximately 30°.

### Medial approach

**Fig. 2:**
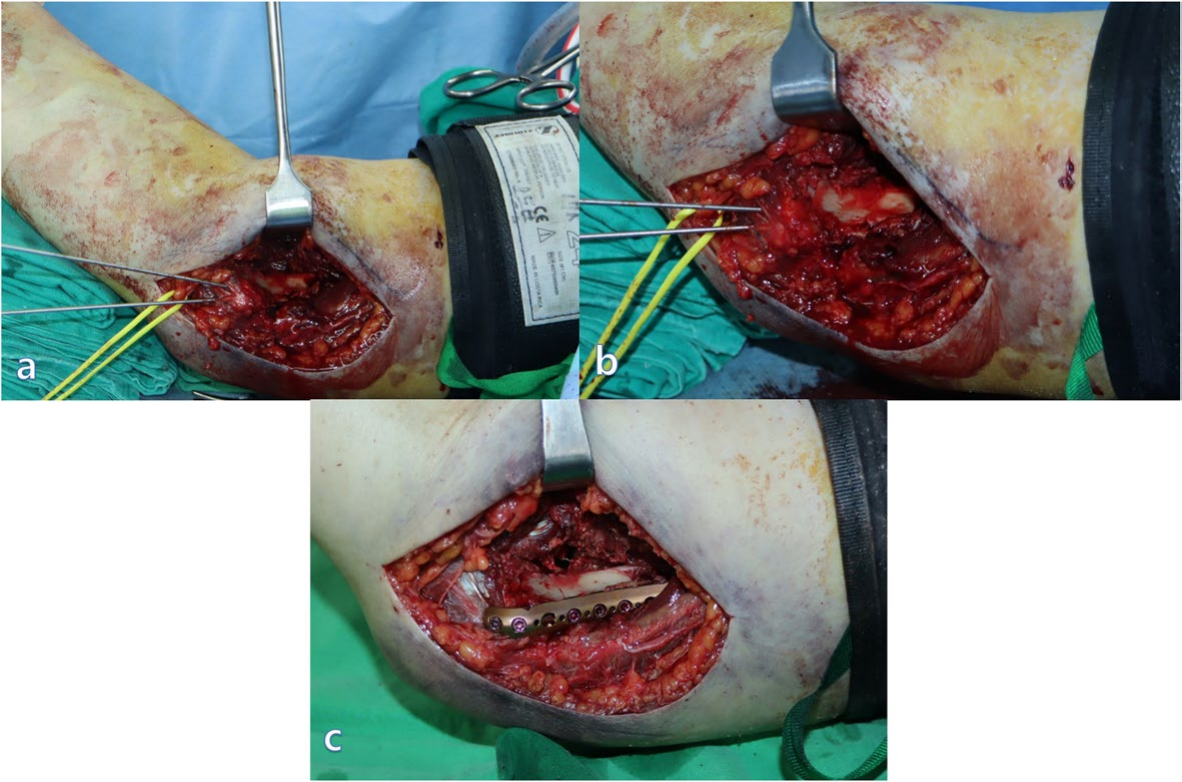
**a** The medial approach was carried out through an incision measuring approximately 8 cm, starting at one finger breadth distally from the tip of the medial epicondyle and proceeding proximally along the medial supracondylar ridge of the humerus toward the axillary line. The elbow was subsequently flexed to approximately 80°; additionally, the biceps and brachialis muscles were retracted anteriorly, and the fracture site was subsequently exposed. **b** The medial column was first reduced, following which one or two 1.6 mm K-wires were inserted for provisional fixation. **c** A short locking compression plate on the medial column

First, the medial approach was performed through an incision measuring approximately 8 cm, starting at one finger breadth distally from the tip of the medial epicondyle and proceeding proximally along the medial supracondylar ridge of the humerus toward the axillary line. We dissected the interval between the brachial muscle and the medial intermuscular septum from the proximal to distal section. The ulnar nerves were palpable posterior to the intermuscular septum; therefore, we proceeded to the distal area without ulnar nerve dissection or releasing from the ulnar nerve groove. The medial and anteromedial surfaces of the distal humerus were exposed by the dissection through the interval. After we approached the elbow joint, we detached the medial intermuscular septum sufficiently from the bone and partially released the muscular origin of the pronator teres muscle to make space for the plate. Subsequently, the anterior joint capsule was incised to expose the articular surface of the trochlea. The elbow was subsequently flexed to approximately 80°, and the biceps and brachialis muscles were retracted anteriorly (Fig. 2a, b). Any hematoma or small fragments in the coronoid fossa could be debrided. The medial column was first reduced, following which one or two 1.6 mm K-wires were inserted for provisional fixation (Fig. 2a, b).

### Lateral approach

**Fig. 3:**
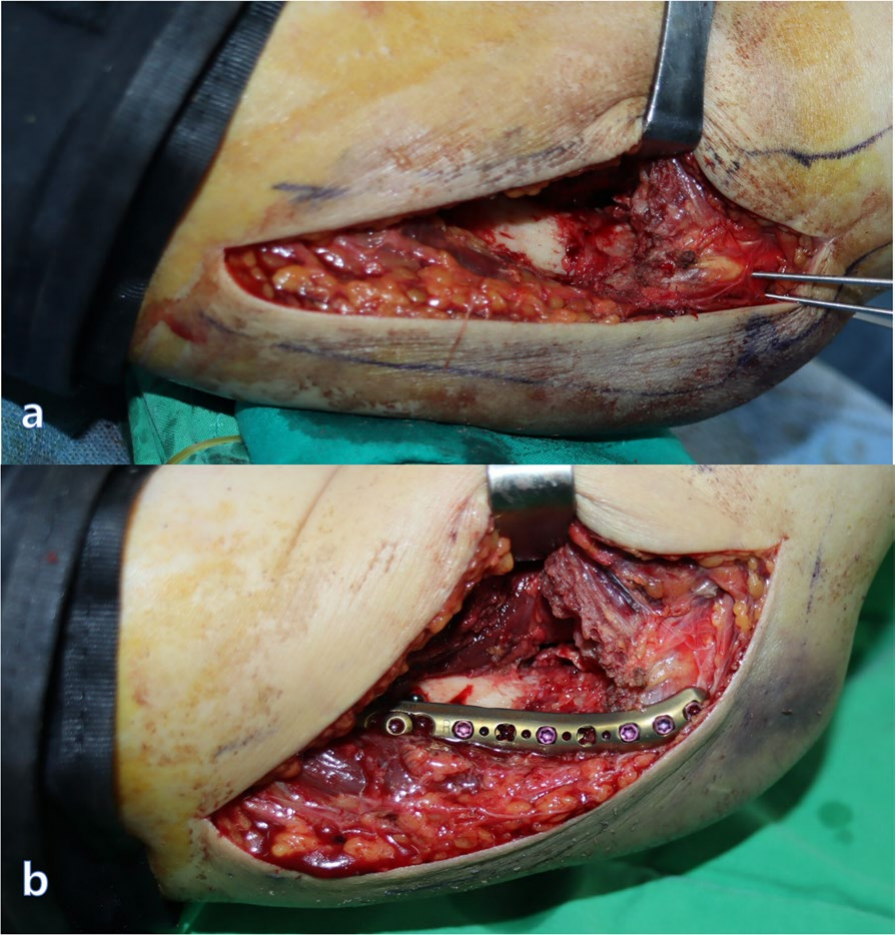
**a** We carefully approached at approximately 10 cm proximal to the lateral epicondyle because the radial nerve pierced the lateral intermuscular septum. Distally, dissection through the interval between the triceps muscle and the origins of the extensor carpi radialis longus and the brachioradialis muscle exposed the lateral border of the humerus. The origin of the brachioradialis was partially released, and the anterior articular surface of the capitulum was exposed. The lateral column was also reduced and temporally fixed using 1.6 mm K-wires. **b** A long locking compression plate was used on the lateral column

**Fig. 4:**
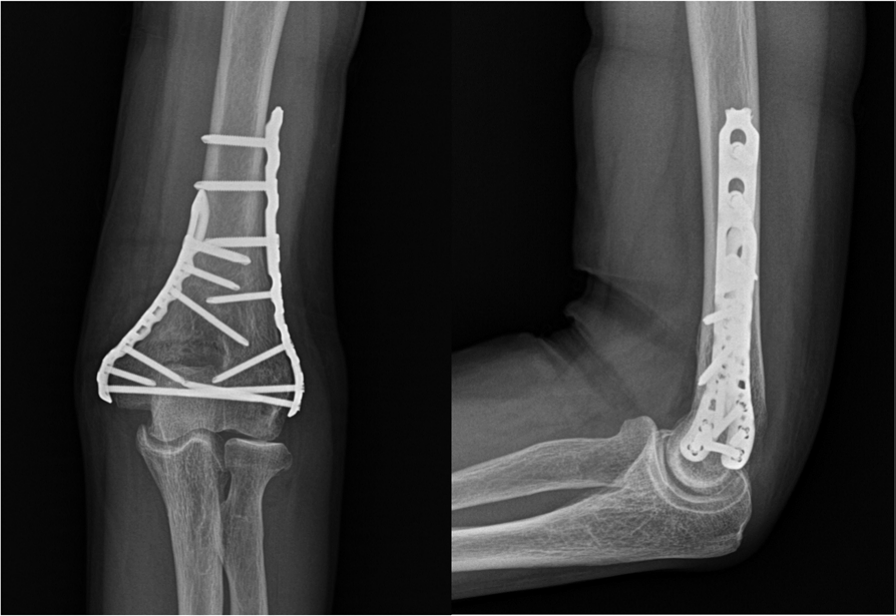
Post-operative radiographs of 75-year-old female patients. The plates are positioned such that the distal screws can be fixed parallel to the anterior surface of the humeral condyles. Two or three screws were fixed to the distal bone fragment on each side; additionally, efforts were made to implant one or more long screws on each side, for the opposite column to gain purchase

A second incision for the lateral approach measured approximately 10 cm, beginning from the distal end of the lateral epicondyle and continuing proximally toward the deltoid tuberosity. The interval between the triceps lateral head and brachialis muscle extended from the distal to the proximal end. We carefully approached at approximately 10 cm proximal to the lateral epicondyle because the radial nerve pierced the lateral intermuscular septum [11]. Distally, dissection through the interval between the triceps muscle and the origins of the extensor carpi radialis longus and the brachioradialis muscle exposed the lateral border of the humerus. The origin of the brachioradialis was partially released, and the anterior articular surface of the capitulum was exposed. The lateral column was also reduced and temporally fixed using 1.6 mm K-wires. In some cases, the simultaneous adjustment of both columns was required (Fig. 3a). The reduction and alignment were subsequently confirmed under direct vision and by using fluoroscopy. Definitive fixation was performed first in the area where additional screws could be fixed to the distal bone fragment. In most cases, to create a difference in the length of the plates, a short locking compression plate (2.7 mm Variable Angle LCP Elbow System, Synthes, Oberdorf, Switzerland) was used on the medial column (Fig. 2c) and a long locking compression plate (3.5 mm Variable Angle LCP Elbow System, Synthes) was used on the lateral column (Fig. 3b). The plates were positioned in such a manner that the distal screws could be fixed parallel to the anterior surface of the humeral condyles. Two or three screws were fixed to the distal bone fragment on each side, and efforts were made to implant one or more long screws on each side, for the opposite column to gain purchase (Fig. 4). The reduction and the length of the screws were checked by the C-arm. Partially released pronator teres and brachioradialis muscle were repaired.

## Post-operative protocol

Postoperatively, the elbow was placed in a bulky non-compressive dressing with a posterior plaster slab to keep the elbow flexed between 40° and 50°. Active-assisted and passive motion was encouraged 3 weeks after the surgical procedure. At 6 weeks, the patients were encouraged to regain ROM of the elbow, and gentle daily activities were permitted. At 3 months, usual activity was allowed.

### Evaluation of radiologic and clinical outcomes

Standardized radiographs (anteroposterior, lateral, internal, and external oblique views) were obtained at 3, 6, and 12 months after surgery and at the last follow-up visit to evaluate for bony union, delayed union, nonunion, heterotopic ossification, or hardware failures. Both the authors and a radiologist reviewed the radiographic results. We evaluated the inter-rater reliability with intraclass correlation coefficients; the obtained value of 0.96 indicated that the inter-rater reliability was excellent. Bone mineral densities were measured using dual energy X-ray absorptiometry (Hologic, Bedford, MA, USA). Osteoporosis was defined using a T-score of ≤ 2.5 according to the criteria of the World Health Organization. Additionally, complications, such as nerve damage and wounds were checked. The operation time was defined as the time from tourniquet inflation to deflation and was assessed by medical records. Blood loss was evaluated as the difference between the preoperative hemoglobin level and the hemoglobin level at 36 h after surgery.

The clinical outcome was assessed based on the VAS, MEPS, DASH, and ROM of the elbow joint, which were collected at the latest follow-up for all the patients. ROM was measured in degrees for flexion, extension, pronation, and supination. We used GraphPad Prism 5.0 (GraphPad, San Diego, California) for statistical analysis of the primary data.

## Results

### Patient characteristics

Three patients were men and 13 patients were women, and the mean age of the patients was 81 years (range, 65–91 years). Nine fractures involved the right humerus (9 dominant), and seven fractures involved the left humerus (1 dominant). The mechanism of injury was a slip down in 12 patients and fall in four patients. According to the AO/OTA classification, 13 patients were classified as type A2.3, two patients were classified as A3.2, and one patient was classified as A3.1. Complete bony union of fractures was achieved in all the patients. The average operation time was 77.8 min (range, 60–100 min). The average difference in preoperative and postoperative hemoglobin was 1.07 g/dL (0.5–1.8 g/dL). We performed internal fixation using parallel plates in 12 cases and using orthogonal plates in 4 cases. The average follow-up was 23.6 months (range, 12–42 months) (Table 1).

**Table 1:** Characteristics and summary of results in 16 cases

Case	Age/Sex	Affected side	Mode of injury	AO/OTA classification	Underlying disease	From injury to Surgery (day)	Follow up period (month)	Time of operation	Blood loss (difference in hemoglobin)	Flexion (degree)	Extension (degree)	ROM (arc)	Pronation (degree)	Supination (degree)	VAS	MEPS	DASH	T-score	Complications
1	85/M	Left	Slip down	A2.3	AA, CKD	67	40	100	1.8	125	0	125	75	80	0	100 (Excellent)	17.2	-3.4	X
2	73/F	Right	Uncertain	A2.3	Dementia	Uncertain	21	90	1	120	-20	100	75	85	3	85 (Good)	24.1	-2.8	X
3	82/F	Right	Slip down	A2.3	Dementia	2	13	70	0.7	110	-5	105	75	85	2	85 (Good)	19.8	-4.3	X
4	81/M	Right	Slip down	A2.3	Arrhythmia, MI	5	32	75	1.9	130	-15	115	60	85	4	70 (Fair)	10.3	-2.3	Loss of reduction in varus
5	66/F	Left	Slip down	A2.3	X	1	25	80	0.9	130	0	130	80	85	3	85 (Good)	19.8	-2.4	X
6	65/F	Right	Slip down	A2.3	X	1	28	90	1.6	130	-20	110	80	60	3	85 (Good)	19.8	-4.4	Stiffness
7	81/F	Left	Slip down	A2.3	HTN	23	19	80	0.7	120	-5	115	85	85	2	85 (Good)	24.1	-2.6	X
8	90/F	Right	Slip down	A2.3	Hypothyroidism	13	17	70	0.7	120	-5	115	80	85	1	85 (Good)	26.7	-4.0	X
9	87/F	Right	Slip down	A2.3	COPD	6	18	65	1	130	-15	115	80	85	1	85 (Good)	18.1	-5.1	X
10	87/F	Right	Slip down	A3.2	Dementia, HTN	3	16	60	0.5	135	-10	125	90	80	2	85 (Good)	33.6	-4.8	Medial skin necrosis
11	85/F	Right	Slip down	A2.3	DM, dementia	Uncertain	23	75	0.8	130	-20	110	90	90	3	100 (Excellent)	13.8	-2.4	X
12	82/F	Right	Slip down	A3.1	DM, HTN	6	15	95	0.9	130	-15	115	75	85	4	70 (Fair)	19.8	-3.9	X
13	91/F	Left	Slip down	A3.2	Dementia, HTN	2	12	90	1.6	125	-10	115	85	90	2	75 (Good)	26.7	-4.2	X
14	83/F	Right	Slip down	A2.3	HTN, hypothyroidism	1	13	75	1.4	130	-15	115	80	85	1	85 (Good)	24.1	-2.7	X
15	74/F	Left	Slip down	A2.3	DM, HTN, polyneuropathy	3	13	60	1	135	-5	130	80	85	1	85 (Good)	9.5	-3.9	X
16	83/M	Left	Slip down	A2.3	Vascular dementia	2	15	70	0.6	130	-15	115	80	85	2	85 (Good)	21.7	-3.8	X

*AO/OTA* Arbeitsgemeinshaft Osteosynthesfragen/Orthopaedic Trauma Association, *VAS* Visual analog scale, *MEPS* Mayo elbow performance score, *DASH* Disabilities of the arm, shoulder and hand, *ROM* Range of motion, *DM* Diabetes mellitus, *HTN* Hypertension, *AA* Aortic aneurysm, *COPD* Chronic obstructive pulmonary disease, *CKD* Chronic kidney disease, *MI* Myocardial infarction

### Radiologic and clinical outcomes

Successful fracture healing was achieved in 15 out of 16 cases with satisfactory bony alignment. Osteoporosis was observed in 13 out of 16 patients, and the average T score was –3.56 (range, –2.3– –5.1).

At the last follow-up evaluation, the mean VAS score for pain was 2.1 (range, 0–6) and the mean MEPS was 84.4 points (range, 70–100 points). Based on the MEPS, 2 patients had an excellent score, 12 had a good score, and 2 had a fair score. The mean DASH score was 20.6 (range, 9.5–33.6). The mean ROM was 116º (range, 100º–130º). The angle of mean flexion was 126.9º (range, 100º–130º); angle of extension, 10.9º (range, 0º–20º); angle of pronation, 83.4º (range, 60º–90º); and the angle of supination was 79.3º (range, 60º–90º) (Table 1).

### Complications

There were three complications (18.8%) in the patients included in the study. Screw migration with loss of reduction in varus was recognized in one case during the early months after fixation. However, the fractures eventually healed without additional surgery. Necrosis of the skin over the ulnar plate was identified 6 months after surgery in one case. Since the fracture was healed, the hardware was removed, and the skin was debrided and re-sutured. One patient suffered approximately 80º of motion restriction (105º of flexion, 35º of extension) at 6 months after surgery. She regained a ROM of about 110º after removal of the instrument without the release of contracture. No complications, such as nerve injuries and heterotopic ossifications were reported in any of the cases.

## Discussion

LTFs of the distal humerus occur mainly in the elderly osteoporotic population; additionally, distal humeral fractures in the elderly population are on the rise [2, 12, 13]. In previous studies, there were several reports that considered total elbow arthroplasty as superior to ORIF in terms of reoperation rate and complications [14, 15]. However, currently, ORIF is the preferred procedure. Parallel and orthogonal plate fixation were widely adopted, while some researchers preferred crisscross-type screw fixation or bicolumnar 90–90 plating [16–18]. Several studies have reported that the results of bi-columnar fixation using a pre-contoured locking plate are similar to or superior to those of total elbow arthroplasty [10, 14, 15, 19]. Goyal et al. [20] reported that in patients who underwent the primary procedure between 2006 and 2016, there was no significant difference in the reoperation risk between total elbow arthroplasty and ORIF. Of all the total elbow arthroplasty reoperations, 6.3% were aseptic revisions, 2.1% were removals of implant, and 1.4% were elbow releases, together comprising approximately 90% of the total reoperations. Conversely, approximately half of the ORIF reoperations (12.1%) involved the removal of instrumentation. This tends to be a minor reoperation, assuming that the fracture is healed at the time the instrumentation is removed. It is now generally accepted that the most favorable outcomes can be provided by surgical reduction through the elbow posterior approach and rigid internal fixation [9]. Since the posterior approach is performed in the lateral or prone position, most surgeries require general anesthesia. Therefore, patients whose state of health precludes general anesthesia may have to choose non-surgical treatments, for which predicting the outcome is difficult.

Ulnar neuropathy poses a unique challenge to the posterior elbow approach, as it can be a product of surgical management and is associated with distal humerus fractures in up to 50% of patients [21]. A meta-analysis by Shearin et al. [22] included 366 patients, of which 187 patients had ulnar nerve in situ decompression and 179 patients had ulnar nerve anterior transposition. The total incidence of ulnar neuropathy was 19.3%, whereas the incidence was 23.5% in the anterior transposed group and 15.3% in the in-situ group. In 2017, Varecka and Myeroff [9] reported 7.2% new postoperative ulnar nerve changes in a pooled analysis of distal humeral fractures, which included 222 patients. Vazquez et al. [23] explained that neuropathy might be the result of several causes, including trauma at the time of the injury, manipulation during splinting, intraoperative manipulation, entrapment in scar tissue, or hardware irritation. Devascularization of the ulnar nerve is considered a cause of ulnar nerve damage [24]. For this reason, in situ decompression has become a more popular surgical treatment option for cubital tunnel syndrome than nerve transposition, which requires devascularization of the ulnar nerve [25, 26]. Unlike other elbow approaches that require devascularization of the ulnar nerve for plate fixation, the bilateral approach described in the present study does not require ulnar nerve dissection; hence, we presumed that damage to the ulnar nerve could be minimized. Furthermore, direct contact with the plate is the main cause of ulnar nerve damage and can be avoided [22]. We performed bi-columnar fixation under brachial plexus anesthesia in the supine position in all 16 patients, including five patients with chronic disease, in whom administration of general anesthesia was difficult. Our approach eliminated the manipulation of the ulnar nerve during surgery by placing the metal plate anterior to the ulnar nerve while preserving the soft tissue liner between them, thus minimizing nerve stimulation. In our retrospective analysis of our small cohort, we found no incidence of ulnar nerve symptoms.

This study evaluates the clinical and radiologic outcomes after a minimum follow-up of 12 months after bi-columnar anatomic locking plate fixation of LTFs in 16 elderly patients. We performed the surgery through a combined medial and lateral approach at the elbow without violating the elbow extension mechanism and without ulnar nerve dissection. The mean age at the time of surgery was 81 years (range, 65–91 years) and patients with poor general health received the surgery in the supine position with brachial plexus anesthesia. In most cases, it was possible to achieve adequate fracture fixation, and our results showed a mean ROM of 10.9° of extension to 126.9° of flexion.

Xie et al. [10] used combined medial and lateral approaches to treat 19 cases of type C (4 cases of C1, 12 cases of C2, and 3 cases of C3) fractures of the distal humerus. They were followed up for an average of 15.8 months, and the mean age of the patients was 44 years (range, 18–79 years). They reported two minor and one major complication; however, no postoperative ulnar nerve changes were reported as in our results. We believe that for non-comminuted fractures of the distal articular surface of the humerus, this approach can be a reasonable option. However, for C3 type intercondylar fractures or comminuted articular surface fractures, it is relatively difficult to reduce and fix the articular fragments under direct vision through this approach; additionally, this approach cannot be converted to olecranon osteotomy to expand the scope of exposure. Therefore, we suggest that this approach should be chosen carefully for C3 fractures.

The main shortcomings of our study were the small sample size and the short follow-up period. These limitations may be due to the low incidence rate of these fractures and as most patients are elderly. The strengths of our study are the exclusion of any other fracture pattern and the inclusion of only an LTF pattern. Additionally, a single surgeon performed all the surgeries, reducing the variability of the results.

In conclusion, LTFs of the distal humerus in the elderly can yield satisfactory results with bi-columnar internal fixation through a combined medial and lateral approach.

## Data Availability

The datasets used and/or analyzed during the current study are available from the corresponding author on reasonable request.

## Abbreviations

AA: Aortic aneurysm
AO/OTA: Arbeitsgemeinshaft Osteosynthesfragen/Orthopaedic Trauma Association
CKD: Chronic kidney disease
COPD: Chronic obstructive pulmonary disease
CT: Computed tomography
DASH: Disabilities of the arm, shoulder, and hand
DM: Diabetes mellitus
HTN: Hypertension
LCP: Locking compression plate
LTF: Low transcondylar fracture
MEPS: Mayo elbow performance score
MI: Myocardial infarction
ORIF: Open reduction and internal fixation
ROM: Range of motion
VAS: Visual analog scale
